# MRI-based radiomics signature is a quantitative prognostic biomarker for nasopharyngeal carcinoma

**DOI:** 10.1038/s41598-019-46985-0

**Published:** 2019-07-18

**Authors:** Xue Ming, Ronald Wihal Oei, Ruiping Zhai, Fangfang Kong, Chengrun Du, Chaosu Hu, Weigang Hu, Zhen Zhang, Hongmei Ying, Jiazhou Wang

**Affiliations:** 1 Department of Radiation Oncology, Fudan University Shanghai Cancer Center, Shanghai, 200032 China; 2 Department of Oncology, Shanghai Medical College, Fudan University, Shanghai, 200032 China

**Keywords:** Cancer imaging, Head and neck cancer

## Abstract

This study aimed to develop prognosis signatures through a radiomics analysis for patients with nasopharyngeal carcinoma (NPC) by their pretreatment diagnosis magnetic resonance imaging (MRI). A total of 208 radiomics features were extracted for each patient from a database of 303 patients. The patients were split into the training and validation cohorts according to their pretreatment diagnosis date. The radiomics feature analysis consisted of cluster analysis and prognosis model analysis for disease free-survival (DFS), overall survival (OS), distant metastasis-free survival (DMFS) and locoregional recurrence-free survival (LRFS). Additionally, two prognosis models using clinical features only and combined radiomics and clinical features were generated to estimate the incremental prognostic value of radiomics features. Patients were clustered by non-negative matrix factorization (NMF) into two groups. It showed high correspondence with patients’ T stage (p < 0.00001) and overall stage information (p < 0.00001) by chi-squared tests. There were significant differences in DFS (p = 0.0052), OS (p = 0.033), and LRFS (p = 0.037) between the two clustered groups but not in DMFS (p = 0.11) by log-rank tests. Radiomics nomograms that incorporated radiomics and clinical features could estimate DFS with the C-index of 0.751 [0.639, 0.863] and OS with the C-index of 0.845 [0.752, 0.939] in the validation cohort. The nomograms improved the prediction accuracy with the C-index value of 0.029 for DFS and 0.107 for OS compared with clinical features only. The DFS and OS radiomics nomograms developed in our study demonstrated the excellent prognostic estimation for NPC patients with a noninvasive way of MRI. The combination of clinical and radiomics features can provide more information for precise treatment decision.

## Introduction

Nasopharyngeal carcinoma (NPC) is an endemic head and neck malignancy in southern China^1^. The main treatment for NPC is radiotherapy^2^. Radiation fractions and prescription doses are related to tumor stages determined by medical imaging at diagnosis^3^. Tumor stages are also important prognostic factors. However, even patients with the same stage may have significantly different treatment responses and prognoses. Increasingly -omics studies of gene and protein patterns^4,5^ are being conducted to provide a deeper understanding of NPC tumor characteristics and to provide decision-making guidance for individualized treatment.

As a routine noninvasive practice, medical imaging can be repeatedly performed in patients for tumor diagnosis and treatment estimation. However, the vast amount of information contained within imaging data need to be further explored. Radiomics is the data mining process that extracts high-throughput features from digital medical images in the region of interest (ROI) with automated algorithms^6^. These quantitative feature have shown validity for predicting tumor treatment responses^7^ and patient prognosis^8,9^.

Magnetic resonance imaging (MRI) has superior soft tissue contrast to computed tomography (CT). The proper selection and combination of a panel of features as a signature has yielded great value for NPC prognosis estimation^10,11^. However, there is a high prevalence of lymph node metastasis among NPC cases^12^. Lymph node status is also important for NPC staging, treatment design and prognosis. To the best of our knowledge, no study has reported an MRI-based radiomics analysis based on both primary and neck lymph node metastatic lesions in NPC patients.

Our study aimed to investigate the prognostic value of the radiomics features of primary and neck lymph node metastasis lesions on MRI and to develop and validate a radiomics signature for estimating NPC prognosis after evaluating feature reliability.

## Results

### Patient characteristics and follow-up

The patient characteristics are shown in Table 1. Fifty-eight patients died or experienced at least one disease relapse during follow-up. The disease relapse and death positivity were 20% and 17.5% in the training and validation cohorts. No differences in patient clinical features were observed between the training and validation cohorts, as shown in Data Supplement Table 1.

**Table 1:** Patient characteristics of the training and validation cohorts.

Characteristics	Training cohort	Validation cohort	Total	DFS	OS	DMFS	LRFS
				5-y	5-y	5-y	5-y
Patients	200	103	303	80.9	88.4	88.8	92.4
Sex							
Male	148 (74)	78 (75.7)	226 (74.6)	77.4	86.7	86.3	92.0
Female	52 (26)	25 (24.3)	77 (25.4)	90.9	93.5	96.1	93.5
T stage							
1	63 (31.5)	21 (20.4)	84 (27.7)	90.5	94.0	94.0	96.4
2	55 (27.5)	27 (26.2)	82 (27.1)	86.6	95.1	91.5	93.9
3	56 (28)	32 (31.1)	88 (29.0)	76.1	83.0	86.4	90.9
4	26 (13)	23 (22.3)	49 (16.2)	63.3	77.6	79.6	85.7
N stage							
0	27 (13.5)	13 (12.6)	40 (13.2)	87.5	90.0	92.5	97.5
1	81 (40.5)	27 (26.2)	108 (35.6)	85.2	88.0	91.7	94.4
2	63 (31.5)	44 (42.7)	107 (35.3)	78.5	87.9	86.9	92.5
3	29 (14.5)	19 (18.5)	48 (15.8)	70.8	89.6	83.3	83.3
Overall stage							
I	13 (6.5)	2 (1.9)	15 (5.0)	100	100	100	100
II	52 (26.0)	14 (13.6)	66 (21.8)	95.5	93.9	97.0	98.5
III	82 (41.0)	48 (46.6)	130 (42.9)	80	87.7	88.5	92.3
IV	53 (26.5)	39 (37.9)	92 (30.4)	68.5	83.7	81.5	87.0
Age							
Median	50	47	48				
Average	49.4	47.7	48.8				
Range	11–80	18–79	11–80				
≤48	96 (48)	57 (55.3)	153 (50.5)	82.4	92.2	88.2	93.5
>48	104 (52)	46 (44.7)	150 (49.5)	79.3	84.7	89.3	91.3
Follow-up time (months)							
Range	5.3–64.2	7.7–39.2	5.3–64.2				
Median	48.9	30.8	40.6				

Note: Data are numbers of patients with percentages in brackets, unless otherwise indicated. The entire 303 patients were stratified according to each clinical feature at respective endpoints. The tumor stage was performed based on the clinical staging of the 7th edition of the American Joint Committee on Cancer (AJCC) TNM staging system.

Abbreviations: 5-y = five-year; p = p value; DFS = disease free-survival; OS = overall survival; DMFS = distant metastasis-free survival; LRFS = locoregional recurrence-free survival.

*p value < 0.05.

### Radiomics analysis on primary lesions

**Figure 1:**
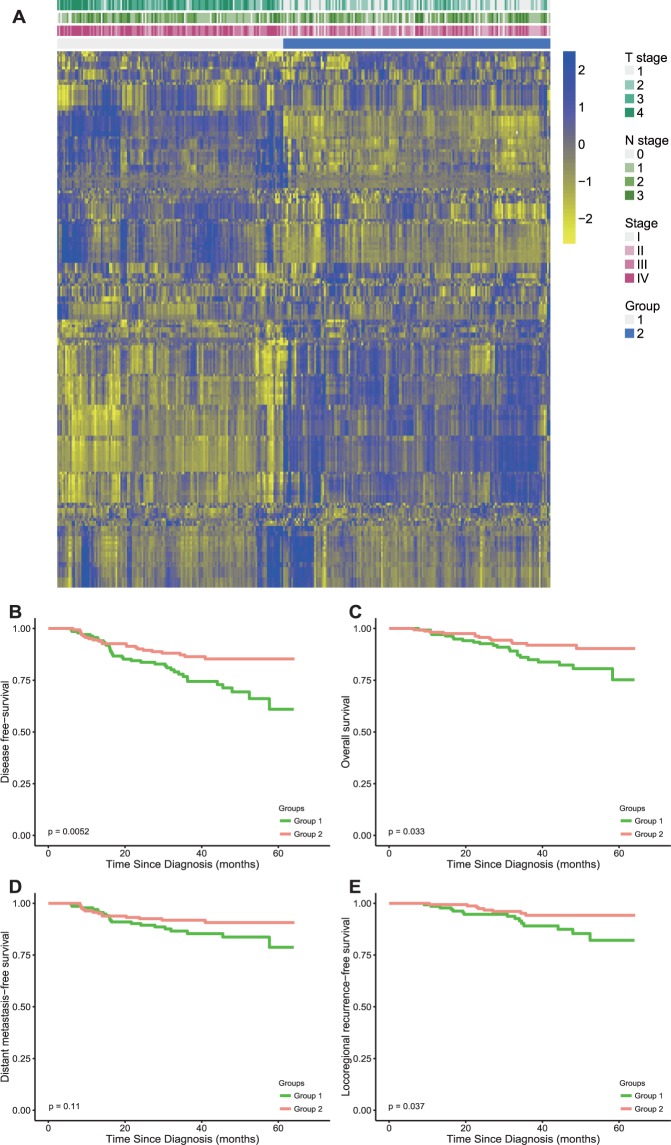
Three hundred and three patients were clustered into two groups according to their radiomics patterns by NMF. (**A**) The clustering results are shown as the heatmap, with 303 patients on the x-axis and the expression of 208 radiomics features on the y-axis. The feature heatmap showed that patients within the same cluster expressed similar radiomics patterns. The clusters showed high correspondence with patients’ T stage (p < 0.00001) and overall stage (p < 0.00001) but not N stage (p = 0.372) according to chi-squared tests. Kaplan-Meier survival curves were constructed for the two NMF clustered groups at each endpoint: (**B**) disease free-survival (DFS); (**C**) overall survival (OS); (**D**) distant metastasis-free survival (DMFS) and (**E**) locoregional recurrence-free survival (LRFS). P values are based on log-rank tests.

In cluster analysis, patients were clustered into two groups of 164 and 139 patients by NMF and the details are provided in Data Supplement Table 2. Clustering results are shown as a heatmap in Fig. 1, with 303 patients on the x-axis and the expression of 208 radiomics features on the y-axis. The feature heatmap showed that patients within the same cluster displayed similar radiomics patterns. The cluster showed high correspondence with patients’ T stage (p < 0.00001) and overall stage (p < 0.00001) but not N stage (p = 0.372) according to chi-squared tests.

Kaplan-Meier survival curves of the two NMF clustered groups for DFS, OS, DMFS and LRFS are also shown in Fig. 1. The log-rank tests showed significant difference in DFS (p = 0.0052), OS (p = 0.033) and LRFS (p = 0.037), but not in DMFS (p = 0.11) between the two groups.

After primary feature selection based on contour reproducibility and nonredundancy, 13 radiomics features were selected according to our criteria for the subsequent construction of prognostic models; the selection results are shown in Data Supplement Table 3.

Details of the prognostic model-building and the respective 10-fold cross-validation results are shown as Data Supplement Tables 4 and 5. The C-index values of all the prognostic models for both training and validation cohorts are presented in Table 2.

**Table 2:** Prognostic model analysis result

	Training			Validation		
	Radiomics	Clinical	Combination	Radiomics	Clinical	Combination
DFS	0.692 [0.618, 0.766]	0.676 [0.596, 0.755]	0.736 [0.665, 0.807]	0.689 [0.563, 0.814]	0.722 [0.618, 0.826]	0.751 [0.639, 0.863]
OS	0.716 [0.613, 0.820]	0.688 [0.589, 0.787]	0.717 [0.624, 0.811]	0.786 [0.644, 0.923]	0.738 [0.555,0.922]	0.845 [0.752,0.939]
DMFS	0.695 [0.588, 0.801]	0.634 [0.526, 0.743]	0.719 [0.607, 0.830]	0.602 [0.433, 0.771]	0.586 [0.437, 0.735]	0.643 [0.481, 0.805]
LRFS	—* —	0.714 [0.590, 0.838]	—* —	— —	0.808 [0.684, 0.932]	—

Note: Data are C-index values with 95% confidence interval in brackets.

*LASSO was unable to generate any radiomics-related model for LRFS in the training cohort in our study.

Abbreviations: DFS = disease free-survival; OS = overall survival; DMFS = distant metastasis-free survival; LRFS = locoregional recurrence-free survival.

The radiomics-based prognostic models were superior in OS prediction to the clinical-based prognostic models in both the training and validation groups. For the models that combined radiomics and clinical features, the C-index was 0.751 [0.639, 0.863] (0.736 [0.665, 0.807]) for DFS and 0.845 [0.752, 0.939] (0.717 [0.624, 0.811]) for OS in the validation (training) cohort. The data also demonstrated the capability of improving the predictive power when combining radiomics and clinical features. That indicated that radiomics features provide additional prognostic information for DFS and OS prediction. All the C-index values of the DMFS models were less than 0.65 in the validation cohort. LASSO-Cox regression failed to identify any radiomics-related features to establish a prognostic model for LRFS.

As the validation of the radiomics signature, the nomogram of the DFS signature and the OS signature are shown in Data Supplement Figs 1 and 2. Five radiomics related features were selected in the DFS signature and two were selected in the OS signature (shown in Data Supplement). The radiomics features of “LL_HIST.kurtosis” and “HL_GLCM.information_Measure_II” contributed to both DFS and OS signatures. “LL_HIST.kurtosis” describes the peak of the tumor image intensity histogram. The gray level co-occurrence matrix (GLCM) represents the joint probability of two particular gray levels within the matrix, and “HL_GLCM.information_Measure_II” describes the inner GLCM linearity dependence after wavelet transformation of the tumor image^13^.

**Figure 2:**
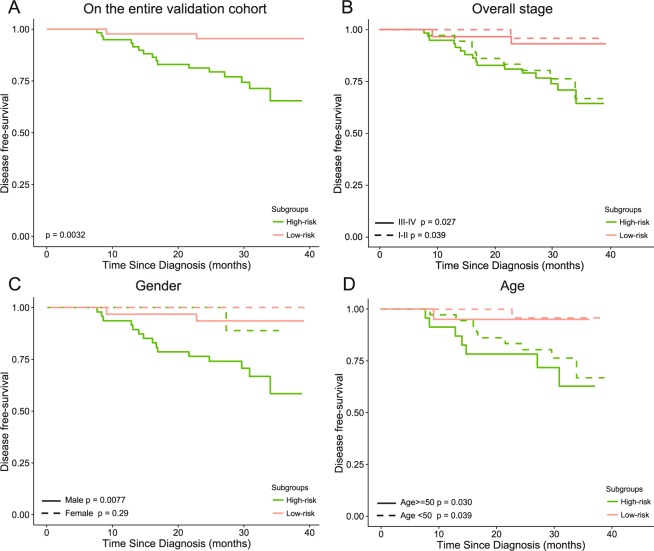
Patients in the validation cohort were divided into a high-risk group and a low-risk group according to their DFS risk score based on a threshold value of −16.66. Kaplan-Meier survival curves of the high- and low-risk patients were constructed on the (**A**) entire validation cohort and in the subgroups stratified by (**B**) overall stage, (**C**) gender and (**D**) age.

The median DFS risk score (the calculation formula is shown in the Data Supplement) was −16.66 in the training cohort. A higher DFS risk score indicated higher DFS risk. The patients in the validation cohort was divided into a high-risk group of 44 patients and a low-risk group of 59 patients according this threshold. The disease progression positivity was 4.5% in the low-risk group versus 27.1% in the high risk group. There were significant differences between the DFS of the high-risk and low-risk patients according to the log-rank tests in the stratified survival analyses, which are shown in Fig. 2.

### Radiomics analysis on neck lymph-node metastatic lesions

Patients were clustered into two groups of 227 and 67 patients by NMF according to their radiomics patterns in lymph node metastatic lesions. The chi-squared tests revealed that the clustering results were significantly related to patients’ N stage (p < 0.0001) and overall stage (p < 0.0001), but not T stage (p = 0.315), as shown in Data Supplement Table 2. Kaplan-Meier survival curves constructed by the NMF clustering results and prognostic model analysis results are shown in Data Supplement Fig. 4 and Data Supplement Table 6. No valuable prognostic information was available through this radiomics analysis focused on neck lymph node metastatic lesions.

## Discussion

MRI has become the main imaging protocol for NPC diagnosis because soft tissue contrast on MRI is superior to that on CT. The MRI-based radiomics features capture tumor characteristics in a quantitative form, and several previous works have proven the utility of such features for diagnosis^14,15^, prediction of treatment responses^16,17^ and prognosis estimation^18^. In this study, the prognostic values of MRI-based radiomics features were explored through different survival analysis methods, and the results demonstrated the prognostic value of these quantitative features.

Radiomics features were primarily selected before prognostic analysis, which focused on contour reproducibility and nonredundancy. Radiomics-related studies usually involve hundreds of features. This high-dimensional feature matrix requires a reasonable test of feature reliability for quality assurance and dimension reduction to avoid redundancy before being integrated as a prognostic signature^19^. Balagurunathan *et al*.^20^ proposed three indicators for radiomics feature selection, which were reproducibility, informativeness and nonredundancy. The features with high reproducibility provided high robustness and were considered to be reliable information for model building^21^.

In the radiomics analysis of primary lesions, the clustering results showed significant correlations with patients’ T stage and overall stage but not N stage. The radiomics features in our study and patients’ T stage are both extensions of primary tumor imaging information. However, MRI-based radiomics features capture the spatial heterogeneity information inside the tumor^22^, while T staging is based on the distance and position of tumor invasion. The relationship between a patient’s radiomics pattern and clinical stage illustrates that the intratumoral heterogeneity generated by cellular and genetic variety can reflect the tumor malignancy level^23^. DMFS is considered more relevant to patient N stage^24^, which is determined based on metastatic lymph nodes. This observation is consistent with the result that the DMFS of patients was not significantly different between the two clustered groups in this study. Patients in the two clustered groups displayed different radiomics patterns and had significantly different DFS, OS, and LRFS in the clustering analysis. These differences illustrate the potential of certain radiomics pattern to supplement the tumor staging system^25^ as precise staging is crucial for NPC treatment design and patient prognosis.

The radiomics signatures for DFS and OS developed in our study demonstrated effective prognostic estimation. Compared with clinical feature-based models, these signatures also showed an improvement for both DFS for OS in their discrimination performances. This improvement demonstrates that spatial heterogeneity inside the tumor provides complementary prognostic information. Stratified survival analyses in the validation cohort, as an application of the DFS radiomics signature, enhanced the accuracy of prognostic prediction after evaluating the patient risk from the signature. Therefore, the DFS signature can help physicians to reasonably adjust the treatment strategy and improve the quality of health care^8^. Two radiomics features, “LL_HIST.kurtosis” and “HL_GLCM.information_Measure_II” were shared in both the DFS and OS radiomics signatures. They captured essential tumor characteristics and might be general prognostic indicators. Parmar *et al*.^26^ studied CT-based radiomics features and found that their radiomics clusters could express the cancer phenotypic characteristics across different cancer types. Patients with higher “LL_HIST.kurtosis” and lower “HL_GLCM.information_Measure_II” presented higher risk of DFS and OS. Both higher “LL_HIST.kurtosis” and lower “HL_GLCM.information_Measure_II” suggest the dense and fine textures of high signals in T1-weighted contrast-enhanced MRI, which are usually indicative of vessels. This agrees with the theory that highly vascular tumors are more likely to progress and lead to poorer prognosis because of the abundant nutrition supplied by the vessels^27,28^. Therefore, integrating MRI-based radiomics with genomics studies^21^ could reveal the underlying mechanisms of tumor growth, such as regulation of angiogenesis.

No additional prognostic information from radiomics features of neck lymph node metastatic lesions was obtained through our approach. Lymph node metastasis lesions generally consist of several smaller positive nodes. These small fragments may cause difficulty in accurate feature calculation because of the limited imaging contrast, which is recognized as partial volume effect^29^. The poor prognostic performance of the features extracted from positive cervical lymph nodes also corresponds to the clinical consensus that particular nodal stations and side distributions are considered to contain more prognostic information^12^.

One limitation of our study was that no radiomics feature was available in LASSO-Cox shrinkage for LRFS estimation, although the clustering results showed a significant difference (p = 0.0037) in patients’ LRFS. This failure was likely caused by an insufficient number of events during follow-up. Therefore, other appropriate feature selection methods are expected for LRFS model building based on larger sample sizes. Moreover, the radiomics signature generated in this study was based on patients from the same clinical center, and external validation is warranted to assess the predictive power^30^. A larger-scale patient dataset for validation can also reduce the false discovery rate of the radiomics based models^31^.

In conclusion, this study demonstrated the utility of radiomics signatures from routine MRI in the evaluation of DFS and OS for NPC patients. Our findings may represent a step toward guiding individualized treatment options and improving health care quality.

## Methods

### Study design

Fudan University Shanghai Cancer Center Institutional Review Board approved this retrospective study and waived the requirement to obtain informed consent. All methods were performed in accordance with the guidelines and regulations of this ethics board.

**Figure 3:**
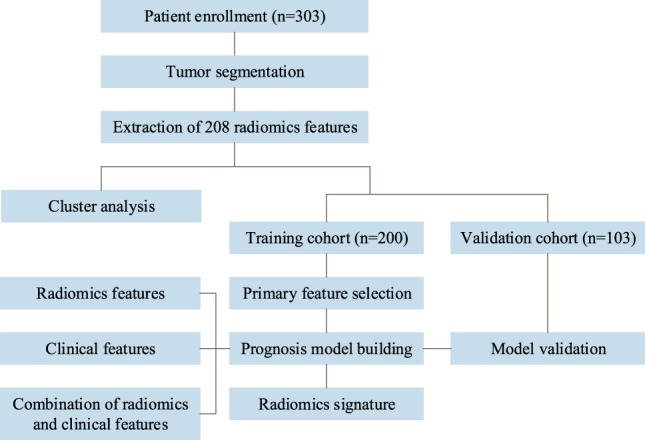
Study work flow. A total of 303 patients were enrolled in this study. The unsupervised cluster analysis was implemented to study the radiomics patterns of the total 303 patients. Primary feature selection, prognostic model building and construction of the radiomics signature were implemented based on the training cohort (n = 200). A validation cohort of 103 patients was used for model validation.

The workflow of this study is shown in Fig. 3. A total of 303 patients were enrolled. After tumor segmentation, 208 radiomics features were separately extracted from the patient’s primary or neck lymph-node metastasis lesions. Then, an unsupervised cluster analysis was implemented to study the radiomics patterns of the 303 patients. After primary feature selection for feature reduction, the prognostic models were built. Disease-free survival (DFS), overall survival (OS), distant metastasis-free survival (DMFS) and locoregional recurrence-free survival (LRFS) were studied as survival outcomes.

### Patients

All patients who underwent MRI scans for NPC pretreatment diagnosis and subsequent treatment at our center from January 2010 to February 2012 were enrolled in this retrospective study (226 men and 77 women; mean age, 48.8 ± 12.7 years; range, 11 to 80 years). Clinical staging of the tumor was performed based on the 7th edition of the American Joint Committee on Cancer (AJCC) TNM staging system^32^. All patients were free of distant metastases (M0) before treatment. The patients’ T stage, N stage, sex, age and digital MRI data were collected from the medical records. The patient inclusion and exclusion criteria are shown in the Data Supplement.

### MRI acquisition, ROI segmentation and radiomics feature extraction methodology

All patients underwent a 1.5 T MRI scan as diagnostic imaging. Transversal contrast-enhanced T1-weighted Digital Imaging and Communications in Medicine (DICOM) images were studied in this radiomics analysis. The detailed MRI acquisition parameters are described in the Data Supplement.

DICOM images of the patients’ MRI were gathered and imported into MIM (version 6.6; MIM Software Inc. Cleveland, OH) for manual segmentation. The primary and lymph-node metastasis lesions of all 303 patients were contoured by an oncologist with 5 years of experience. Nineteen patients were randomly selected and their lesions were recontoured by another oncologist for the contour reproducibility study. All segmentation was checked by a senior oncologist experienced in NPC.

After tumor segmentation, 208 radiomics features were extracted from the contrast-enhanced T1-weighted MRI data by an in-house algorithm called “QIAT” in MATLAB R2015a (The MathWorks Inc, Natick, MA) for each patient. The radiomics features included image intensity histogram analysis (10), texture analysis (31), wavelet analysis^21^ (164) and fractal analysis^33^ (3). The feature extraction methodology has been described in detail in the Data Supplement. The primary and lymph-node metastatic lesions served as two different ROIs and the radiomics features were separately extracted from the two.

### Cluster analysis

Non-negative matrix factorization (NMF) is widely used for the clustering of high-dimensional datasets in computational biology^34^. After feature extraction, NMF was used to cluster 303 patients into two groups according to their radiomics feature patterns. Details are provided in the Data Supplement. Then, the chi-squared test was used to study the relationship between tumor stage and the clustering results. Kaplan-Meier survival curves for DFS, OS, DMFS and LRFS were also constructed for the two clustered groups. The log-rank test was used to study the significance of the differences among the clustered groups for each endpoint.

### Prognostic model analysis

Patients were first segregated according to their pretreatment diagnosis date into a training cohort and a validation cohort. The training cohort of 200 patients was used to build and optimize the prognostic model, and a validation cohort of 103 people was used to validate the estimation power of the model. The Mann-Whitney U test was used to study the differences in clinical features between the training and validation cohorts.

Primary feature selection on contour reproducibility and nonredundancy was implemented before building the prognostic models. To study contour reproducibility, 19 randomly selected patients in the training cohort were contoured independently by two oncologists. The intra-class correlation coefficient (ICC2) was calculated by the radiomics features extracted from both segmentations to evaluate the segmentation reproducibility^35^. Features with ICC2 > 0.8^36^ were selected. Redundant features with pairwise correlation were removed by the “findCorrelation” function with a cutoff of 0.5 using the “caret” packages in R for nonredundancy selection.

After primary feature selection, a prognostic model for each endpoint was built by the least absolute shrinkage and selection operator (LASSO) Cox regression^37^ using the training cohort. When building a model, LASSO shrinks the algebraic sum of the feature coefficients into a penalty parameter “lambda”. Therefore, some feature coefficients were reduced into zero to achieve the minimum lambda. The leave-one-out cross validation (LOOCV) method was used to optimize the penalty parameter “lambda” for LASSO because LOOCV achieves a thorough data mining and provides an almost unbiased estimator^38^. Additionally, the widely recommended “lambda.1se”^39^ was used as the penalty parameter of LASSO to establish a concise prediction model. Further 10-fold cross-validation was also performed to achieve a steady result.

To study the incremental prognostic value of radiomics features, two other prognostic models were built using the same methods: one based on clinical features (T stage, N stage, age and sex) only and the other based on a combination of both radiomics and clinical features. The Harrell concordance index (C-index)^40^ and 95% confidence intervals (CIs) for the respective models were calculated to assess the estimation performance of the models in both the training and validation cohorts.

### Construction and validation of the radiomics signature

The radiomics signature for each endpoint combined both clinical features and the radiomics features extracted from the primary lesions. The signatures were generated by LASSO for the training cohort after summing the radiomics score. The radiomics score was calculated by summing up the non-zero radiomics features according to their coefficient weights. A nomogram was constructed for respective signatures based on the validation cohort.

The DFS risk score of the patients in the training cohort were evaluated according to the DFS radiomics signature. The median value of their DFS risk score was applied as a threshold to divide the patients on the training cohort into a high-risk group or low-risk group. Stratified analyses were implemented to compare the high-risk and low-risk patients’ DFS in various subgroups.

All statistical analyses were conducted with R software (version 3.3.1; http://www.Rproject.org), and all related analysis packages are listed in the Data Supplement Table 7. Statistical significance levels are indicated by two-sided p values with α set at 0.05.

## Supplementary information

Supplement
