# Supplementary material for: MRI-based radiomics signature is a quantitative prognostic biomarker for nasopharyngeal carcinoma
Source: Sci Rep. 2019 Jul 18;9:10412. doi: 10.1038/s41598-019-46985-0 (PMC6639299; doi:10.1038/s41598-019-46985-0)
Supplement: Supplementary file 1 — Supplement [file 41598_2019_46985_MOESM1_ESM.pdf]

MRI-based radiomics signature is a quantitative prognostic biomarker for nasopharyngeal carcinoma

Title

Authors and affiliations.

Xue Ming[1,2*],Ronald Wihal Oei[1,2*],Ruiping Zhai[1,2], Fangfang Kong[1,2], Chengrun Du[1,2], Chaosu Hu[1,2], Weigang Hu[1,2], Zhen Zhang[1,2], Hongmei Ying[1,2†], Jiazhou Wang[1,2†]

1. Department of Radiation Oncology, Fudan University Shanghai Cancer Center, Shanghai 200032, China
2. Department of Oncology, Shanghai Medical College, Fudan University, Shanghai 200032, China

*Xue Ming and Ronald Wihal Oei contributed equally to the work.

† Hongmei Ying and Jiazhou Wang are both the corresponding authors.

Corresponding author. Hongmei Ying and Jiazhou Wang are both the corresponding authors.

Name: Hongmei Ying

Mailing address: Department of Radiation Oncology, Fudan University Shanghai Cancer Center, Department of Oncology, Shanghai Medical College, Fudan University, Shanghai 200032, China,

No.270 Dongan Road, Xu Hui District, Shanghai, China

Telephone: 021-64175590

Fax number: 021-64174774

Email address: yinghongmei@zoho.com.cn

Name: Jiazhou Wang

Mailing address: Department of Radiation Oncology, Fudan University Shanghai Cancer Center, Department of Oncology, Shanghai Medical College, Fudan University, Shanghai 200032, China,

No.270 Dongan Road, Xu Hui District, Shanghai, China

Telephone: 021-64175590

Fax number: 021-64174774

Email address: wjiazhou@gmail.com

Data Supplement:

1) the Mann-Whitney U test on patients' clinical parameter difference

Supplementary Table 1. Results of the Mann-Whitney U test in the training and validation cohorts

	T stage	N stage	Age	Gender	Overall stage
p value	0.11	0.11	0.22	0.67	0.2

2) Results of the NMF clustered groups

Supplementary Table 2. Results of the NMF clustered groups

Results of the cluster based on primary lesions					
T stage	T1	T2	T3	T4	Total
group1	78	51	33	2	164
group2	6	31	55	47	139
N stage	N0	N1	N2	N3	Total
group1	26	54	56	28	164
group2	14	54	51	20	139

Overall stage	I	II	III	IV	Total
group1	15	48	72	29	164
group2	0	18	58	63	139
Results of the cluster based on lymph-node metastasis lesions					
T stage	T1	T2	T3	T4	Total
group1	24	24	16	12	76
group2	60	58	72	37	227
N stage	N0	N1	N2	N3	Total
group1	40	27	3	6	76
group2	0	81	104	42	227
Overall stage	I	II	III	IV	Total
group1	15	27	16	18	76
group2	0	39	114	74	227

Three hundred and three patients were clustered into two groups by NMF according to their radiomics patterns, and the stage information in each group is listed in the supplementary table 2.

3) Primary feature selection results

Supplementary Table 3. Primary feature selection results

Primary feature selection results based on primary lesions			
Radiomics feature	Radiomics group	Filter transformation	Description
LL_GLCM.Information_Measures_II	GLCM	Wavelet LL	To describe the linear-dependence inside GLCM
HL_GLCM.Information_Measures_II	GLCM	Wavelet HL	
HH_GLCM.Correlation_Original	GLCM	Wavelet HH	To describe the element similarity inside GLCM
HL_GLRLMS.SRHGE	GLRLMS	Wavelet HL	To describe the joint distribution of high gray-level values in short run length
LL_HIST.kurtosis	Statics	Wavelet LL	To describe the peakness of gray level probability histogram
LH_HIST.kurtosis	Statics	Wavelet LH	
HL_HIST.kurtosis	Statics	Wavelet HL	
HH_HIST.kurtosis	Statics	Wavelet HH	

LL_HIST.skewness	Statics	Wavelet LL	To describe the symmetry of gray level probability histogram
LH_HIST.skewness	Statics	Wavelet LH	
LL_HIST.min	Statics	Wavelet LL	To measure the minimum gray level value
LL_HIST.mean	Statics	Wavelet LL	To measure the average gray level value
HH_HIST.median	Statics	Wavelet HH	To measure the median gray level value
Primary feature selection results based on lymph-node metastasis lesions			
Radiomics feature	Radiomics group	Filter transformation	Description
LL_HIST.skewness	Statics	Wavelet LL	To describe the symmetry of gray level probability histogram
LH_HIST.skewness	Statics	Wavelet LH	
HL_HIST.skewness	Statics	Wavelet HL	
HH_HIST.skewness	Statics	Wavelet HH	
LL_HIST.min	Statics	Wavelet LL	To measure the minimum gray level value
HH_HIST.mean	Statics	Wavelet HH	To measure the average gray level value

FRACTAL_DIMENSION.Mass	Fractal	LoG 1	To describe the number of non-zero elements within the cycle of specific center and radius

*L=low, H=high

*LoG=Laplacian of Gaussian

*GLCM=gray level co-occurrence matrix, which represents the joint probability of two particular gray levels within the matrix.

*GLRLMS=gray level run-length matrices, which calculate the number of elements with the same gray-level value in specific direction length.

*SRHGE=Short Run High Gray Level Emphasis, which measures the joint distribution of high gray-level values in short run length.

*After reproducibility study and redundancy selection, thirteen radiomics features based on primary lesions and seven radiomics features based on lymph-node metastasis lesions were selected according to our criteria.

4) The details of the prognosis models based on radiomics features extracted from

the primary lesions

Supplementary Table 4. The details of the prognosis models based on radiomics features extracted from the primary lesions

**Endpoint**	**DFS**		
Model	**Radiomics**	**Clinical**	**Combined (the DFS radiomics signature)**
Feature	LL_GLCM.Information_Measures_II	T_stage	LL_GLCM.Information_Measures_II
coefficients	-2.057	0.3675	-16.54
Feature	HL_GLCM.Information_Measures_II	N_stage	HL_GLCM.Information_Measures_II
coefficients	-6.011	0.093012	-3.860
Feature	LL_HIST.mean	Age	LL_HIST.mean
coefficients	0.0001794	0.001543	7.68E-05
Feature	LL_HIST.kurtosis	Gender	LL_HIST.kurtosis
coefficients	0.4142	-0.1706	0.1818
Feature	HH_HIST.median		HH_HIST.median
coefficients	-1.222		-2.113
Feature			T_stage
coefficients			0.3309
Feature			N_stage
coefficients			0.05431
Feature			Age
coefficients			0.005721
Feature			Gender
coefficients			-0.2041
**Endpoint**	**OS**		
Model	**Radiomics**	**Clinical**	**Combined (the OS radiomics signature)**
Feature	HL_GLCM.Information_Measures_II	T_stage	HL_GLCM.Information_Measures_II

coefficients	-4.237	0.2364	-3.653
Feature	LL_HIST.skewness	Age	LL_HIST.kurtosis
coefficients	0.5880	0.01405	0.3785
Feature	LL_HIST.kurtosis		T_stage
coefficients	0.4420		0.07689
Feature			Age
coefficients			0.006962
**Endpoint**	**DMFS**		
Model	**Radiomics**	**Clinical**	**Combined**
Feature	LL_GLCM.Information_Measures_II	T_stage	LL_GLCM.Information_Measures_II
coefficients	-2.387	0.1686	-13.50
Feature	HL_GLCM.Information_Measures_II		HL_GLCM.Information_Measures_II
coefficients	-4.640		-4.345
Feature	LL_HIST.skewness		LL_HIST.skewness
coefficients	0.2985		0.4256
Feature	LL_HIST.kurtosis		LL_HIST.kurtosis
coefficients	0.4014		0.2631
Feature			LL_HIST.min
coefficients			0.002395
Feature			T_stage
coefficients			0.2187
**Endpoint**	**LRFS**		
Model	**Radiomics**	**Clinical**	**Combined**
Feature	-	T_stage	-
coefficients	-	0.3394	-
Feature		N_stage	
coefficients		0.3632	
Feature		Age	
coefficients		0.01074	

The models were all generated by LASSO. LASSO shrinks the regression coefficients into a penalty parameter, therefore the coefficient of some feature was zero in the optimized model. In supplementary Table 4, the selected features and the respective coefficients of the models (radiomics-based, clinical based and combined) were listed for each endpoint. T- and N-stages were coded on a linear scale and sex was coded as: 0=male, 1=female before building the models.

5) The results of the model performance with 10-fold cross validation

Supplementary Table 5. Prognostic model analysis result

	Radiomics	Clinical	Combination
DFS	0.674<br>[0.586, 0.761]	0.657<br>[0.567, 0.748]	0.710<br>[0.611, 0.810]
OS	0.694<br>[0.600, 0.788]	0.669<br>[0.546, 0.791]	0.700<br>[0.626, 0.774]
DMFS	0.669<br>[0.534, 0.805]	0.623<br>[0.510, 0.735]	0.689<br>[0.600, 0.778]

LRFS	-*	0.679	-*
	-	[0.581, 0.776]	-

Note: Data are C-index values with 95% confidence interval in brackets.

Abbreviations: DFS=disease free-survival; OS=overall survival; DMFS=distant metastasis-free survival; LRFS= locoregional recurrence-free survival

6) The DFS and OS radiomics signature

The DFS and OS radiomics signature was generated by summing up the selected radiomics features by their respective coefficients in the combined models. The detailed definition was shown as followed:

The DFS radiomics score=$-16.54 \times \mathrm{LL\_GLCM.Information\_Measures\_II}$

$$-3.860 \times \mathrm{HL\_GLCM.Information\_Measures\_II}$$

$$+\ 7.680\mathrm{E}-05 \times \mathrm{LL\_HIST.mean}$$

$$+\ 0.1818 \times \mathrm{LL\_HIST.kurtosis}$$

$$-\ 2.113 \times \mathrm{HH\_HIST.median}$$

The OS radiomics score= $-3.653 \times \mathrm{HL\_GLCM.Information\_Measures\_II}$

$$+0.3785 \times \mathrm{LL\_HIST.kurtosis}$$

7) The DFS risk score:

The patients were evaluated for their DFS risk score according to the DFS radiomics signature. The DFS risk score is calculated by summing up all the features inside the signature by a linear combination. The calculation formula is shown as below:

The DFS risk score=the $\mathrm{DFS\ radiomics\ score} + 0.3309 \times \mathrm{T\_stage}$

$$+0.05431 \times \mathrm{N\_stage} + 0.005721 \times \mathrm{Age} - 0.2041 \times \mathrm{Gender}$$

T- and N-stages were coded on a linear scale and sex was coded as: 0=male, 1=female.

8) The nomogram of the DFS radiomics signature:

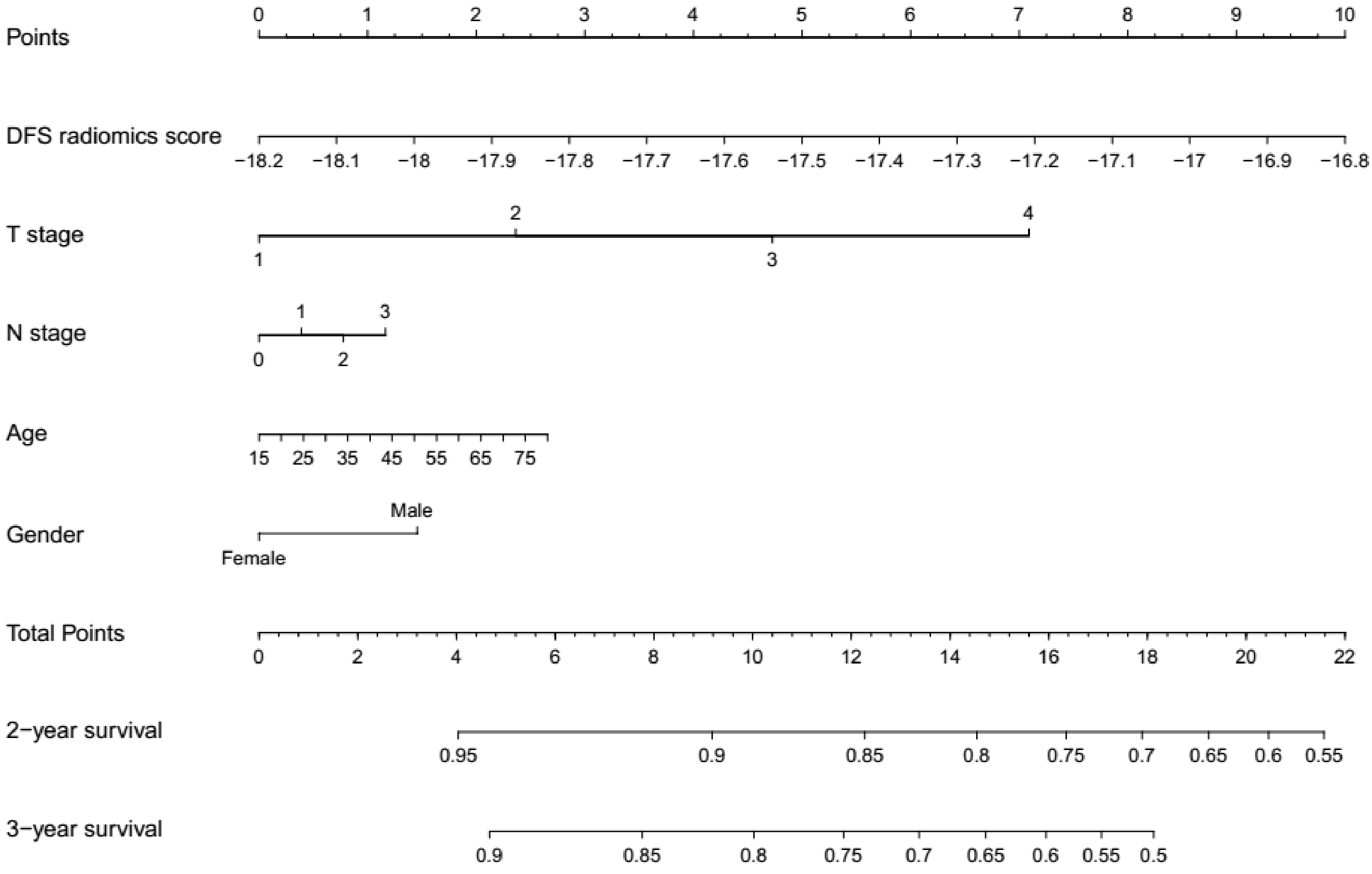

Supplementary Figure 1. The nomogram of the DFS radiomics signature. The nomogram was developed in the validation cohort and aided in the estimation of two- and three-year disease-free survival (DFS) probability of patients with nasopharyngeal carcinoma. The respective score according the patients' T stage is determined by drawing a straight line upward to the "Points" axis. This procedure is repeated for the other parameters, and the scores for all the parameters are then

summed. The total sum is then located on the Total Point axis, and a straight line is drawn downward to determine the patient's DFS probability.

9) The nomogram of the OS radiomics signature:

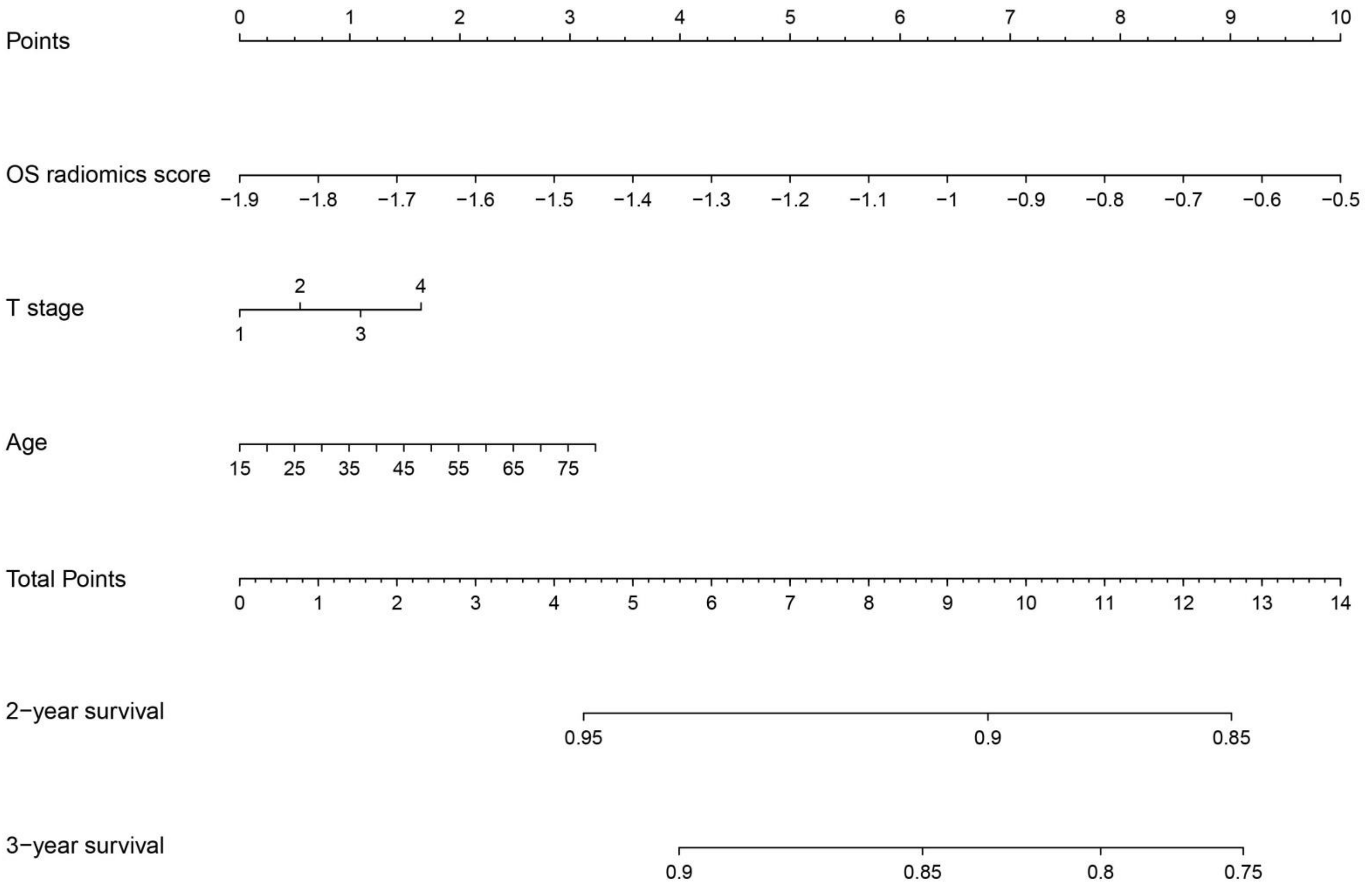

Supplementary Figure 2. The nomogram of the OS radiomics signature. The nomogram was developed in the validation cohort and it helped the users to estimate the probability of two- and three-year overall survival (OS) of a patient with nasopharyngeal carcinoma. Draw a straight line upward to the "Points" axis to

determine the respective score according the patient’s T stage. Repeat the procedure for the other parameters and sum up the scores for all the parameters. Locate the final sum on the Total Point axis. Draw a line straight down to determine the patient’s probability of OS.

10) **The Results of the cluster analysis on the radiomics features extracted from the lymph-node metastasis lesions**

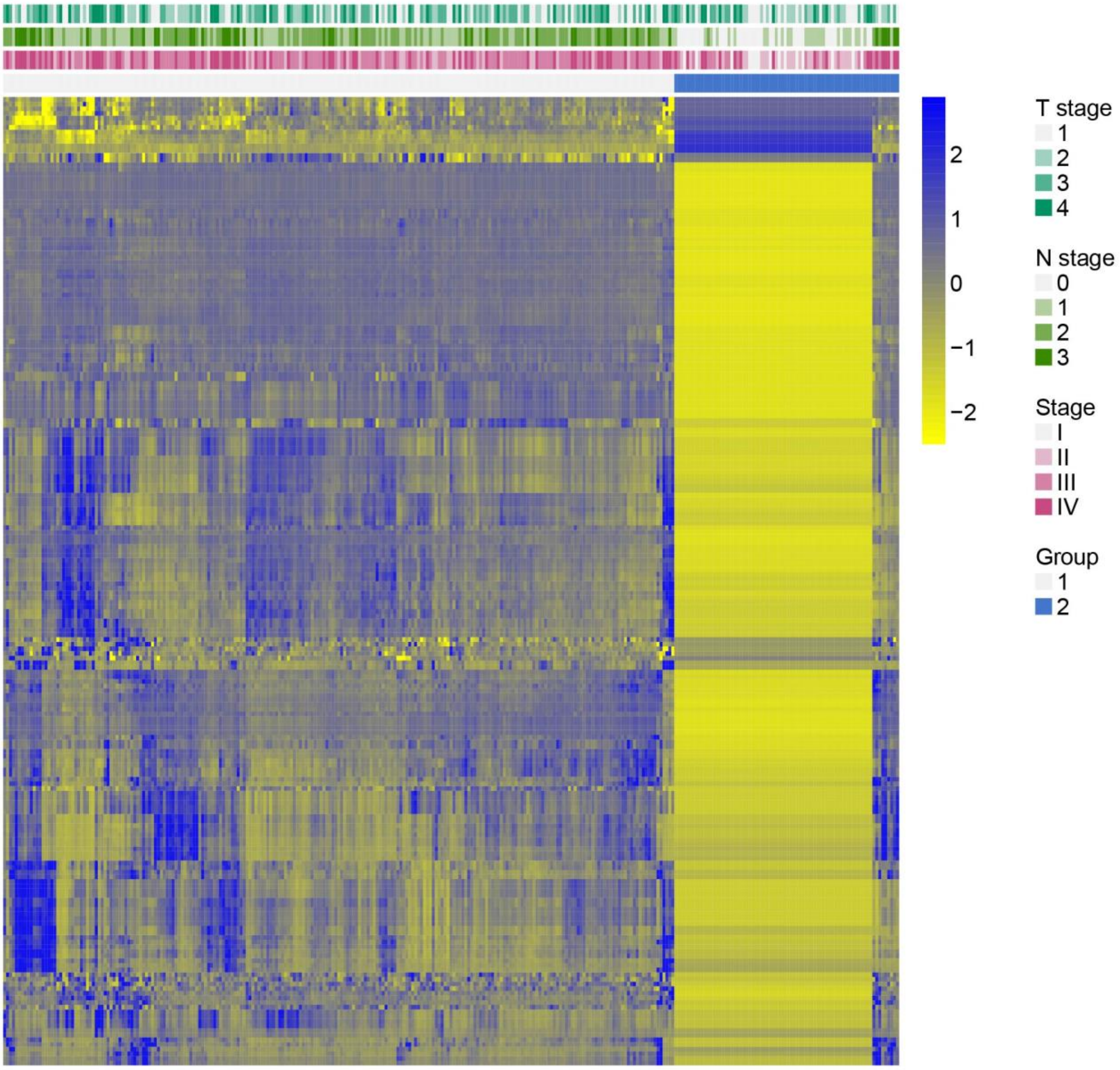

Supplementary Figure 3. Radiomics features clustered heatmap. Clustering results were based on lymph-node metastasis lesions and shown as the heatmap, with 303 patients on the x-axis and their radiomics expression on the y-axis. The clustering

results showed a significant relationship with patients' N stage (p<0.0001) and overall stage (p<0.0001), but not T stage (p=0.315) by chi-squared tests.

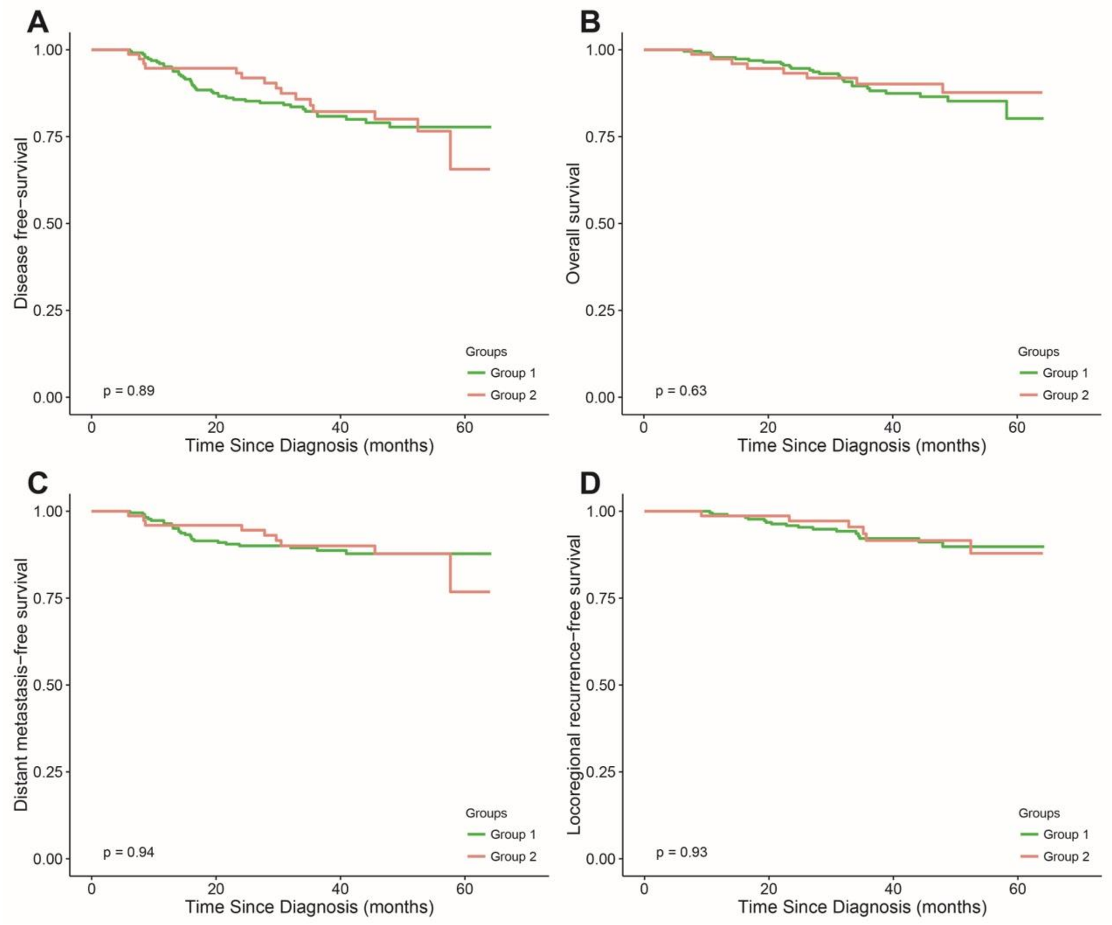

Supplementary Figure 4. Prognosis performance of NMF clustered groups. Clustering results were based on lymph-node metastasis lesions of 303 patients. Kaplan-Meier survival curves were constructed by the two clustered groups for A) disease free-survival (DFS); B) overall survival (OS); C) distant metastasis-free survival (DMFS) and D) locoregional recurrence-free survival (LRFS). No

significance difference was observed in two NMF clustered groups on DFS (p=0.89), OS (p=0.63), DMFS (p=0.94) and LRFS (p=0.93).

11) The prognosis analysis on the radiomics features extracted from the lymph-node metastasis lesions

Supplementary Table 6. Prognosis model analysis result

	Training			Validation		
	Radiomics	Clinical	Combination	Radiomics	Clinical	Combination
DFS	0.652	0.676	0.648	0.552	0.722	0.653
	[0.570,0.735]	[0.596, 0.755]	[0.566,0.729]	[0.422,0.681]	[0.618, 0.826]	[0.538,0.768]
OS	-	0.688	0.713	-	0.738	0.774
	-	[0.589, 0.787]	[0.607,0.809]	-	[0.555,0.922]	[0.574,0.974]
DMFS	0.632	0.634	0.671	0.520	0.586	0.598
	[0.513,0.750]	[0.526, 0.743]	[0.557,0.785]	[0.384,0.656]	[0.437, 0.735]	[0.442,0.755]
LRFS	-	0.714	-	-	0.808	-
	-	[0.590, 0.838]	-	-	[0.684, 0.932]	-

*DFS=disease free-survival; OS=overall survival; DMFS=distant metastasis-free survival; LRFS= locoregional recurrence-free survival

The prognosis model analysis results are shown as Supplementary Table 6 as the C-index values of respective prognostic models in both training and validation cohorts. Ninety-five percentage confidence intervals (CI) for respective models in brackets. Prognostic models only based on radiomics features failed to estimate DFS and DMFS since their C-index on the validation cohort were not significantly better than random. Also LASSO failed to select any radiomics feature for prognosis model building on OS and LRFS.

12) Patient inclusion and exclusion criteria:

The inclusion criteria:

- **a)** Patients were histologically confirmed primary nasopharyngeal carcinoma (pNPC).
- **b)** Patients were free of distant metastases at diagnosis.
- **c)** Patients underwent 1.5T MRI scans at our center before treatment.

d) DICOM of the T1-weighted contrast-enhanced MRI for both nasopharyngeal and neck scans of the patients were available.

e) Clinical data were available, including the age, sex and their staging information according to the 7th AJCC TNM stage edition.

The exclusion criteria:

a) Patients received any treatment for NPC before their first nasopharyngeal MRI scan.

b) DICOM of the patients' MRI were not available.

c) Clinical data was incomplete.

13) MRI acquisition parameters:

MRI was conducted with a 1.5 T GE Signa scanner (GE, Milwaukee,WI), using an 8-channel phased-array joint coil and Gd-DTPA as the contrast-enhanced agent. The acquisition parameters of transversal T1-weighted contrast-enhanced MRI were as follows: imaging protocol: fast spoiled gradient recalled echo-based scanning (FSPGR), repetition time (TR):185-215 ms, echo time (TE): 1.9-2.5 ms, slice

thickness: 6mm, flip angle: 80˚. The in-plane resolution is 0.468-0.523 mm and matrix size is 512×512.

14) Feature extraction methodology:

In this study, 208 radiomics features were extracted from the primary (lymph-node metastasis) lesion of a single patient. The radiomics features included four groups: image intensity histogram analysis (10), texture analysis (31), wavelet analysis[1] (164) and fractal analysis[2] (3).

Group One: Image intensity histogram analysis:

The image intensity histogram analysis was based on the grey level histogram of the medical images. From the grey level histogram, ten features were extracted including: mean, median, variance, entropy, skewness, kurtosis, min, max, standard deviation and range. The mathematics definition of these histogram analysis features was described by Aerts et.al[1].

Group Two: Texture analysis:

The texture analysis aimed to study the spatial distribution of the voxels with similar grey levels in the images. Grey level co-occurrence (GLCM) matrices[3] and gray level run-length (GLRLM) matrices[4] were used to describing the texture patterns in this study. Twenty GLCM-based features and 11 GLRLM-based features were extracted from the primary (lymph-node metastatic) lesion of a patient.

A) GLCM-based features:

Contrast, correlation, energy, homogeneity 1, homogeneity 2, variance, standard deviation, dissimilarity, entropy, sum average, difference average, sum variance, difference variance, sum entropy, difference entropy, information measure of correlation 1, information measure of correlation 2, maximal correlation coefficient, and autocorrelation were extracted from a patient's lesion. The mathematic definition of the features were shown by Haralick[3,5].

B) GLRLM based features:

Short Run Emphasis (SRE), Long run Emphasis (LRE), Gray Level Non-Uniformity (GLN), Run Length Non-Uniformity (RLN), Run Percentage (RP), Low Gray Level Run Emphasis (LGLRE), High Gray Level Run Emphasis (HGLRE),

Short Run Low Gray Level Emphasis (SRLGLE), Short Run High Gray Level Emphasis (SRHGLE), Long Run Low Gray Level Emphasis (LRLGLE) and Long Run High Gray Level Emphasis (LRHGLE) were extracted from a patient's lesion. The mathematics definition of the above ten GLRLM based features were described by Aerts et al.[1].

Group Three: Wavelet analysis：

The original images are decomposed by the wavelet transformation into the combination of high and low frequency signals. In this study, each slice of the images is decomposed by two kinds of wavelet transformation, the high-pass or low-pass filters [6] along the two directions. Therefore, the original image X is transformed into X_{LL} (low-pass filter along both directions), X_{HL}, X_{LH} (low-pass filter along on direction and high-pass filter along another direction) and X_{HH} (high-pass filter along both direction).

After the wavelet transformation, the four decomposition images were performed histogram analysis and texture analysis and generated 4×41 wavelet based radiomics features.

Group Four: Fractal analysis

Fractal analysis aims to study the patterns of the similar elements in different dimensions and scales. In this study, three fractal based radiomics features were extracted from a patients' lesion, including box counting dimension[6], Minkowsky dimension[6] and mass dimension[6].

In this study, histogram based radiomics features were based on the histogram of the grey levels in all MRI slices. Texture based and wavelet based radiomics feature (155) was the average value of the respective mathematic calculation results on each slice. For example, when extracting the radiomics feature of "kurtosis" in histogram analysis, if a patient has twenty slices of MRI, a row value will be calculated according to the mathematics definition of "kurtosis" on each one of the twenty slices. And the radiomics feature of "kurtosis" extracted from this patient is the average

value of the above twenty row values on the respective twenty slices. Previous works on reproducibility of the radiomics features by Hu et al. [7]showed that the average type of radiomics features presented a better robustness on the segmentation reproducibility. The fractal based features was extracted from the slice with the maximum area of tumor segmentation of a patient.

15) NMF details:

In this study, three hundred and three patients were clustered into two groups by NMF, and NMF preformed 500 runs to achieve a stable clustering results.

16) Packages in R:

All the packages in R program used in this study are listed in the supplementary Table 7.

Supplementary Table 7. The R packages used in this study

Packages name	Author	Function
survival	Terry Therneau	to perform survival analysis

NMF	Original definition:<br>D D Lee and HS Seung[8]<br>Port to R: Renaud Gaujoux	to perform NMF clustering according to the patients radiomics features pattern
ggplot2	Hadley Wickham<br>Winston Chang	to illustrate the Kaplan-Meier surviving curves according to the NMF clustered results in each endpoint
scales	Hadley Wickham	
survminer	Alboukadel Kassambara, Marcin Kosinski, Przemyslaw Biecek and Scheipl Fabian	
irr	Matthias Gamer	to calculate the intra-class correlation coefficient (ICC2) in reproducibility study
caret	Max Kuhn	to remove the redundant features with pair-wise correlation
glmnet	Jerome Friedman, Trevor Hastie, Noah Simon and Rob Tibshirani	to build prognosis models through LASSO Cox regression

Hmisc	Frank Harrell, Charles Dupont	to calculate the C-index of prediction model
rsm	Russell V. Lenth	to illustrate the nomogram based on radiomics signature of DFS and OS

Reference:

1 Aerts, H. J. *et al.* Decoding tumour phenotype by noninvasive imaging using a quantitative radiomics approach. *Nature communications* **5**, 4006, doi:10.1038/ncomms5006 (2014).

2 Pentland, A. P. Fractal-Based Description of Natural Scenes. *IEEE Transactions on Pattern Analysis and Machine Intelligence* **PAMI-6**, 661-674, doi:10.1109/TPAMI.1984.4767591 (1984).

3 Haralick, R. M., Shanmugam, K. S. & Dinstein, I. Textural Features for Image Classification. *IEEE Transactions on Systems, Man, and Cybernetics* **3**, 610-621 (1973).

4 Galloway, M. M. Texture Analysis using Gray Level Run Lengths. *Computer Graphics Image Processing* **4**, 172-179 (1975).

5 Haralick, R. M. & Shapiro, L. G. Computer and Robot Vision - Volume 1. *IEEE Robotics & Automation Magazine* **18**, 121-122 (1992).

6 Haidekker, M. *Advanced Biomedical Image Analysis*. (John Wiley & Sons, 2010).

7 Hu, P. *et al.* Reproducibility with repeat CT in radiomics study for rectal cancer. *Oncotarget* **7**, 71440-71446, doi:10.18632/oncotarget.12199 (2016).

8 Lee, D. D. & Seung, H. S. Learning the parts of objects by non-negative matrix factorization. *Nature* **401**, 788, doi:10.1038/44565 (1999).
